# Fungal-associated NO is involved in the regulation of oxidative stress during rehydration in lichen symbiosis

**DOI:** 10.1186/1471-2180-10-297

**Published:** 2010-11-22

**Authors:** Myriam Catalá, Francisco Gasulla, Ana E Pradas del Real, Francisco García-Breijo, Jose Reig-Armiñana, Eva Barreno

**Affiliations:** 1 Universidad Rey Juan Carlos, Biología Celular, Dpto. Biología y Geología, (ESCET), Madrid, Spain; 2 Universitat de València, Botánica & ICBIBE-Jardí Botànic, Fac. CC. Biológicas, C/Dr. Moliner 50. 46100-Burjassot. Valencia, Spain; 3 U. Politécnica de Valencia. Dpto. Ecosistemas Agroforestales. Camino de Vera s/n. 46022-Valencia, Spain

## Abstract

**Background:**

Reactive oxygen species (ROS) are normally produced in respiratory and photosynthetic electron chains and their production is enhanced during desiccation/rehydration. Nitric oxide (NO) is a ubiquitous and multifaceted molecule involved in cell signaling and abiotic stress. Lichens are poikilohydrous organisms that can survive continuous cycles of desiccation and rehydration. Although the production of ROS and NO was recently demonstrated during lichen rehydration, the functions of these compounds are unknown. The aim of this study was to analyze the role of NO during rehydration of the lichen *Ramalina farinacea *(L.) Ach., its isolated photobiont partner *Trebouxia *sp. and *Asterochloris erici *(Ahmadjian) Skaloud et Peksa (SAG 32.85 = UTEX 911).

**Results:**

Rehydration of *R. farinacea *caused the release of ROS and NO evidenced by the fluorescent probes DCFH_2_-DA and DAN respectively. However, a minimum in lipid peroxidation (MDA) was observed 2 h post-rehydration. The inhibition of NO in lichen thalli with c-PTIO resulted in increases in both ROS production and lipid peroxidation, which now peaked at 3 h, together with decreases in chlorophyll autofluorescence and algal photobleaching upon confocal laser incidence. *Trebouxia sp*. photobionts generate peaks of NO-endproducts in suspension and show high rates of photobleaching and ROS production under NO inhibition which also caused a significant decrease in photosynthetic activity of *A. erici *axenic cultures, probably due to the higher levels of photo-oxidative stress.

**Conclusions:**

Mycobiont derived NO has an important role in the regulation of oxidative stress and in the photo-oxidative protection of photobionts in lichen thalli. The results point to the importance of NO in the early stages of lichen rehydration.

## Background

Lichens are symbiogenetic organisms composed of fungi (mycobionts) and their photosynthetic partners (photobionts). They are poikilohydrous, subject to repeated desiccation/rehydration cycles, and able to survive in extreme, frequently very dry environments, such as deserts or the arctic tundra. Reactive oxygen species (ROS) are known to be a major cause of damage during desiccation, especially in photosynthetic organisms [1]. In some species, rehydration provokes an extracellular oxidative burst (reviewed in [2]) and it has been shown that the status of the antioxidant glutathione (GSH) is correlated with the ability of lichens to tolerate desiccation [3-5]. Furthermore, there is evidence of effective communication between mycobionts and photobionts, in which one partner up-regulates the antioxidant system of the other, endowing the symbiotic association with an important adaptive advantage and evolutionary success [6]. Nonetheless, much remains to be learned about lichen metabolism of ROS during dehydration/rehydration cycles, since it has been recently reported that classical antioxidant mechanisms play a limited role in the strategies that facilitate transition of photobionts to the desiccated state [7].

Reactive oxygen species are produced in the respiratory and photosynthetic electron chains of many organisms. In photosynthetic organisms, the production of ROS is enhanced during desiccation and/or rehydration because carbon fixation is impaired, whereas chlorophyll electrons continue to be excited. ROS result from the uncontrolled donation of electrons from electron transport chains in chloroplasts and mitochondria to molecular oxygen, initiating an indiscriminate chain reaction. If antioxidant defenses are overcome by ROS production, the uncontrolled free radicals cause widespread cellular damage by provoking protein alterations, lipid peroxidation, and the formation of DNA adducts [8].

The bioactive gas nitric oxide (NO) has multiple biological functions in a very broad range of organisms. These functions include signal transduction, cell death, transport, basic metabolism, ROS production and degradation [9,10], among others (reviewed in [11]). It is well-known that NO exerts both pro-oxidant and antioxidant effects, depending on the ambient redox status, the presence of other reactants, and the nature of the reaction (for a review of the antioxidant actions of NO, see [12]). In plants, ROS and reactive nitrogen species have been shown to be involved in the defensive response of plants to biotic or abiotic stresses such as pathogens [13], drought [14], and air pollutants or UV-B radiation [15]. In the latter study, the authors found support for the hypothesis that NO reactive species, together with the glutathione system, play a key role in the coordination of gene expression during plant symbiosis. NO has been postulated as one of the first antioxidant mechanisms to have evolved in aerobic cells [16,17]. This idea builds on the work of Feelisch and Martin [18], who suggested a role for NO in both the early evolution of aerobic cells and in symbiotic relationships involving NO efficacy in neutralizing ROS. In addition, NO is involved in the abiotic stress response of green algae such as *Chlorella pyrenoidosa *Pringsheim, by reducing the damage produced by photo-oxidative stress [19].

The first work that focused on NO production in lichens was published in 2005, by Weissman and co-workers [20], who carried out a microscopy study of *Ramalina lacera *(With.) J.R. Laundon. These authors described the occurrence of intracellular oxidative stress during rehydration together with the release of NO by the mycobiont, but not by the photobiont. We have recently reported evidence that NO is involved in oxidative stress in lichens exposed to the oxidative pollutant cumene hydroperoxide [21]. However, there have been no further studies on NO function, or on the occurrence of NO in other lichens.

Real-time imaging of cellular function *in vivo *and of cell/tissue localization can be achieved with high sensitivity and specificity by using fluorescent probes together with fluorescence and confocal microscopy. For example, following entry of the probe DCFH_2_-DA into the cell it is converted by intracellular esterases to DCFH_2_, which upon oxidation by free radicals, mainly ^•^OH, CO_3_^•-^, NO_2_^•^, and thyl radicals (such as GS^•^), yields the fluorescent product (DCF) (reviewed by [22]).

Nitrogen oxide is produced at low concentrations and has a short half-life, which makes it difficult to detect in vivo. Interest in NO, due to its ubiquity and physiological relevance, has therefore led to the generation of several techniques for measuring its production. For example, the rapid reaction of 2,3-diaminonaphthalene (DAN) with NO to form the fluorescent product 1-(H)-naphthotriazole (NAT) is the basis for a very sensitive analytical method to measure NO production. DAN does not react directly with NO and therefore does not inhibit its actions. The high sensitivity of this technique allows its use in the quantification of NO production in living cells [23-25]. However, perhaps the most commonly employed methods for the analysis of NO in aqueous solutions is by measuring NO_2_^- ^using the Griess reagent [23]. Alternatively, inhibitors of NO function can also be used to understand the physiological roles of this molecule. Carboxy-PTIO (c-PTIO) is a water-soluble and stable free radical that reacts stoichiometrically with NO. *In vivo*, c-PTIO inhibits the physiological effects mediated by NO, whereas *in vitro *it can be used to quantitate NO levels by ESR spectrometry [11].

The lichen *Ramalina farinacea *(L.) Ach. is a widespread species with large environmental tolerance. This green-greyish lichen is a fruticose, pendulous, epiphytic species that is very common in Mediterranean sclerophyllous oak forests. It lives on a great variety of substrates and different habitats such as plant bark, decomposing wood and rocks [26]. In the Iberian Peninsula it occurs at all altitudes, more frequently in areas with regular fogs being absent in maritime habitats. It shows especial preference for places with a high atmospheric humidity. This lichen is the *Ramalina *species with lower sensitivity to SO_2 _and is considered as toxitolerant [27].

The aim of this work is to investigate the release and role of NO in the oxidative stress caused by rehydration in the lichen *Ramalina farinacea *(L.) Ach. NO and ROS specific fluorescent probes will be used to morphologically localize these molecules *in vivo *with fluorescence and confocal microscopy. Furthermore, ROS kinetics and chlorophyll autofluorescence will be recorded during the first minutes after rehydration. Lipid peroxidation and NO-endproducts will be quantified at different time points. NO especific inhibitor c-PTIO will be used in order to elucidate NO functions. Likewise, NO production and relation with photosynthesis will be studied in different models of isolated photobionts: *Ramalina farinacea *(L.) Ach. isolated *Trebouxia sp*. photobionts, and in *Asterochloris erici *(Ahmadjian) Skaloud et Peksa, SAG 32.85 = UTEX 911.

## Methods

### Chemicals

The chemicals 2,6-di-tert-buthyl-4-methylphenol trichloroacetic acid (BHT), 2-thiobarbituric acid (TBA), 1,1,3,3, tetraethoxypropane (TEP), cumene hydroperoxide 88% (CP), and bisbenzimide H (Hoechst) were provided by Sigma Aldrich Química S.A (Tres Cantos, Spain); 2,7-dichlorodihydrofluorescein diacetate (DCFH_2_-DA), hydrochloric acid (HCl) and ethanol (etOH) were purchased from Panreac Química S.A.U (Barcelona, Spain); 2-(4-carboxyphenyl)-4,4,5,5-tetramethylimidazoline-1-oxyl-3-oxide potassium salt (cPTIO) and 2,3-diaminonaphthalene (DAN) were from Invitrogen S.A (El Prat de Llobregat, Spain); and Triton X-100 was from VWR Prolabo (Barcelona, Spain).

### Lichen material

*Ramalina farinacea *(L.) Ach. was collected in the air-dried state from *Quercus rotundifolia *Lam. at Sierra de El Toro (Castellón, Spain; 39°54'16"N, 0°48'22"W). Samples were maintained in a silica gel atmosphere during 24 h and frozen at -20°C until the experiment, 1 month after collection.

### Epifluorescence probes

2,7-Dichlorodihydrofluorescein diacetate (DCFH_2_-DA) was used as probe in the detection of ROS (DCF, λ_exc _= 504 nm, λ_em _= 524 nm). DCFH_2_-DA is not appreciably oxidized to the fluorescent state without prior hydrolysis inside the cell.

2,3-Diaminonaphthalene (DAN) reacts with the nitrosonium cation that forms spontaneously from NO to yield the fluorescent product 1H-naphthotriazole which emits blue fluorescence (λ_exc _= 375 nm, λ_em _= 425 nm). Since the selectivity of DAN for the nitrosonium cation is high, NO can be detected without the inhibition of its function [25].

### Fluorometric Kinetics of Free Radical Production and Chlorophyll Autofluorescence

Dry fragments of lichen thalli were placed in black flat bottom 96 multiwell plates and kept at -20°C until use. One of the plates was rehydrated with deionised water 24 h before the experiment and kept at 17°C, PAR 35 μmol m^-2 ^s^-1 ^16 h photoperiod.

Both dry and hydrated lichens were submerged during 5 minutes in deionised water 10 μM DCFH_2_-DA with or without c-PTIO 200 μM. The excess of solution was eliminated and the kinetics of DCF and chlorophyll emitted fluorescence were simultaneously measured in a SPECTRAFluor Plus microplate reader (Tecan Group Ltd., Männedorf, Switzerland). Excitation of both substances was performed at λ_exc _485 nm, emission of DCF fluorescence was recorded at λ_em _535 nm and chlorophyll autofluorescence at λ_em _635 nm, during one hour. Twelve replicates were analyzed by treatment and all values are referred to the weight of sample.

### Microscopy

Fragments of lichen thalli were rehydrated for 5 min with either deionized water or 200 μM c-PTIO, and the corresponding fluorescence probe (10 μM DCFH_2_-DA or/and 200 μM DAN). The samples were then placed in a freezing microtome (CM 1325; Leica, Germany) and cut in sections of 30 microns. The slices were washed with deionized water and mounted on slides prior to their observation by fluorescence microscopy (OLYMPUS Provis AX 70 fluorescence microscope) or confocal laser scanning microscopy (TCS Leica SP Confocal Laser Scanner Microscope, Leica, Heidelberg, Germany) at the SCSIE (UVEG, Valencia).

### Isolated photobionts of *Ramalina farinacea*

The photobiont *R. farinacea *(*Trebouxia *sp.) was isolated following the protocol described by Gasulla et al. [28]. Basically, it involves homogenization of lichen thalli (from 15 mg to 2 g), a one-step centrifugation through Percoll (r), followed by washing with Tween 20 and sonication. Algae were grown in 3N Bold's basal medium (BBM3N) containing 10 g casein and 20 g glucose per liter [29] with a 16:8 h light:dark photoperiod and at a temperature of 15°C. The medium was changed every 2 weeks and the concentration of algae set at 10^5 ^cells/ml.

### Physiology of photosynthesis

An axenic strain of the lichen photobiont *Asterochloris erici *(Ahmadjian) *Skaloud et Peksa *(SAG 32.85 = UTEX 911) was used for this study. Algae were grown on cellulose-acetate discs on agar BBM3N containing 10 g casein and 20 g glucose per liter [29,30]. Cultures were maintained at 20°C under a 12 h photoperiod with 30 μmol m^-2^s^-1 ^white-light illumination.

After 21 days, the discs were removed from the culture medium and dried in a closed container with a saturated solution of ammonium nitrate (R.H. 62%), and maintained under culturing conditions. The samples remained in the dried state for 24 h, were then rehydrated with distilled water or 200 μM c-PTIO and returned to culture conditions for 24 h.

*In vivo *chlorophyll *a *fluorescence was measured with a modulated light fluorometer (PAM-2000, Walz, Effeltrich, Germany). The samples were kept in the dark for 30 min and the minimum (dark) fluorescence yield (F_o_) measured after excitation of the algae with a weak measuring beam from a light-emitting diode. The maximum fluorescence yield (F_m_) was determined with an 800 ms saturating pulse of white light (SP, 8000 μmol m^-2 ^s^-1^). Variable fluorescence (F_v_) was calculated as F_m_-F_o_, and the maximum quantum yield of photosystem II (PSII) as F_v_/F_m_. The samples were allowed to re-adapt in the dark for 2 min, after which actinic light (AL, 200 μmol m^-2 ^s^-1^, unless otherwise stated) was switched on, and SPs were applied at 1 min intervals to determine: (1) the maximum fluorescence yield during actinic illumination (F'_m_), (2) the level of modulated fluorescence during a brief (3 s) interruption of actinic illumination in the presence of 6 μmol m^-2 ^s^-1 ^far red (FR, 730 nm) light (F'_o_), and (3) steady-state chlorophyll *a *fluorescence yield after 11 pulses (F_s_). Photochemical quenching (qP), and the quantum efficiency of PSII photochemistry (Ф_PSII_) were estimated following the methods of Genty et al. [31] and Kramer et al. [32].

### Measurement of malondialdehyde

Lipid peroxidation was evaluated as malondialdehyde (MDA) by the method of Reilly and Aust [33], modified as described below [34,35]. Working standards were made by diluting a 2 mM stock solution of the malondialdehyde precursor TEP with 80% ethanol supplemented with 2% of the antioxidant BHT to suppress the decomposition of lipid peroxides during the assay. Working concentrations of 0-50 μM were prepared for the lichens and 0-8 μM for the algae.

Lichen thalli were homogenized on ice with 1 ml of deionized water and centrifuged at 16,060 × *g *for 10 min. Supernatants were frozen at -20°C for NO_x _analysis, and the pellets resuspended in 500 μl ethanol-BHT. Algae were homogenized directly in 500 μl of ethanol-BHT with glass fragments (approx. 1 mm diameter) and strong vortexing for 30 min. Subsequently, 900 μM of TBA (2.57 × 10^-2^M), TCA (9.18 × 10^-1^M), and HCl (3.20 M) working solution was added to each sample and to the standards. The samples and standards were vortexed in a Vortex Labnet ×100 for 5 min at 3,000 rpm and then placed in a 70°C water bath for 30 min. Afterwards, the samples and standards were vortexed again, cooled on ice, and centrifuged at 10,060 × *g *for 10 min.

The absorbance of supernatants was measured at 532 nm (*A*_532_) in a Spectronic Genesys8 spectrophotometer. The absorbance at 600 nm (*A*_600_) was then measured and this value was subtracted from the *A*_532 _to eliminate the interferences of soluble sugars in the samples [35].

### NO end-products determination

To estimate NO generation, NO oxidation end-products (nitrate and nitrite) were measured in the soluble fraction of the samples using a Skalar autoanalyzer, model SAN++. The automated determination of nitrate and nitrite is based on the cadmium reduction method: the sample is passed through a column containing granulated copper-cadmium to reduce nitrate to nitrite. The nitrite (that originally present plus that obtained from the reduction of nitrate) concentration is determined by its diazotization with sulfanilamide followed by coupling with N-(1-naphthyl)ethylenediamine dihydrochloride to form a highly colored azo dye, the absorbance of which is measured at 540 nm. This is the most commonly used method to analyze NO production and is known as the Griess reaction [23].

### Statistics

At least three samples for each treatment and each incubation time were prepared. Four assays were carried out on four different days for the lichens and on three different days for the algae. Data were analyzed for significance with Student's *t*-test or by ANOVA.

## Results

**Figure 1:**
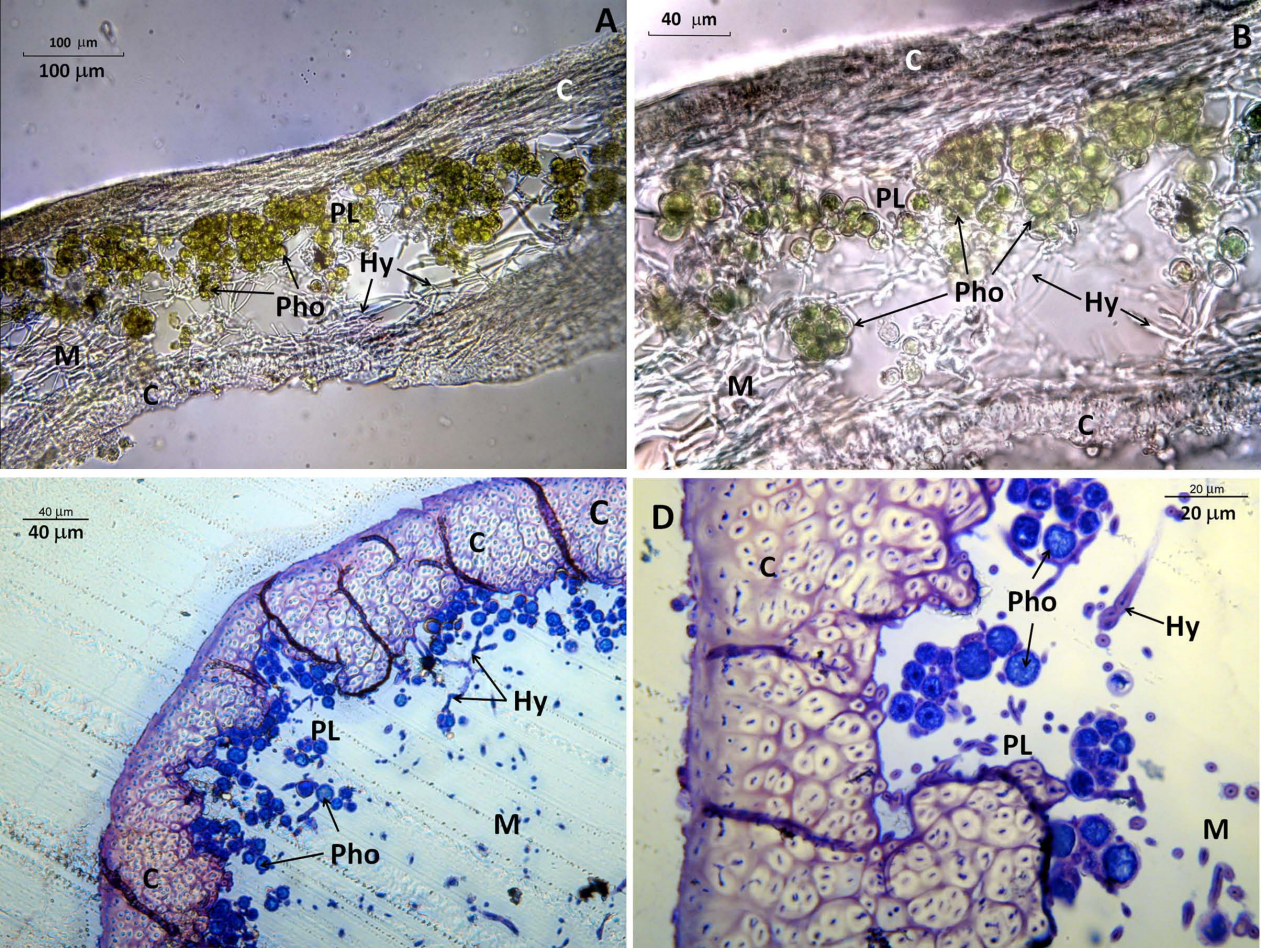
**Anatomy of *Ramalina farinacea***. Thalli of *R. farinacea*: **A, B **Bright-field microscopy of slices cut in a freezing microtome (magnification 200× and 1000×, respectively); **C, D **ultramicrotome survey sections (10 μm) stained with toluidine blue (magnification 200× and 1000×, respectively). C cortex, PL photobiont layer, Pho photobiont, M medulla, Hy fungal hyphae

Bright-field micrographs showing the general anatomy of *Ramalina farinacea *are presented in Figure 1. The photobiont layer is located in the medulla and is surrounded by dispersed fungal hyphae, which become densely packed in the cortex of the lichen.

### ROS generation, chlorophyll autofluorescence and lipid peroxidation during lichen rehydration

Although several works have described an extracellular oxidative burst during rehydration in some lichen species, virtually nothing is known about intracellular ROS production and its relationship to abiotic stress. In order to determine whether intracellular ROS release follows the rehydration of *R. farinacea *thalli, 10 μM of the fluorescent probe DFCH_2_-DA was added to the deionized water used for rehydration. The samples were observed by fluorescence and confocal microscopy 3-4 h after rehydration.

**Figure 2:**
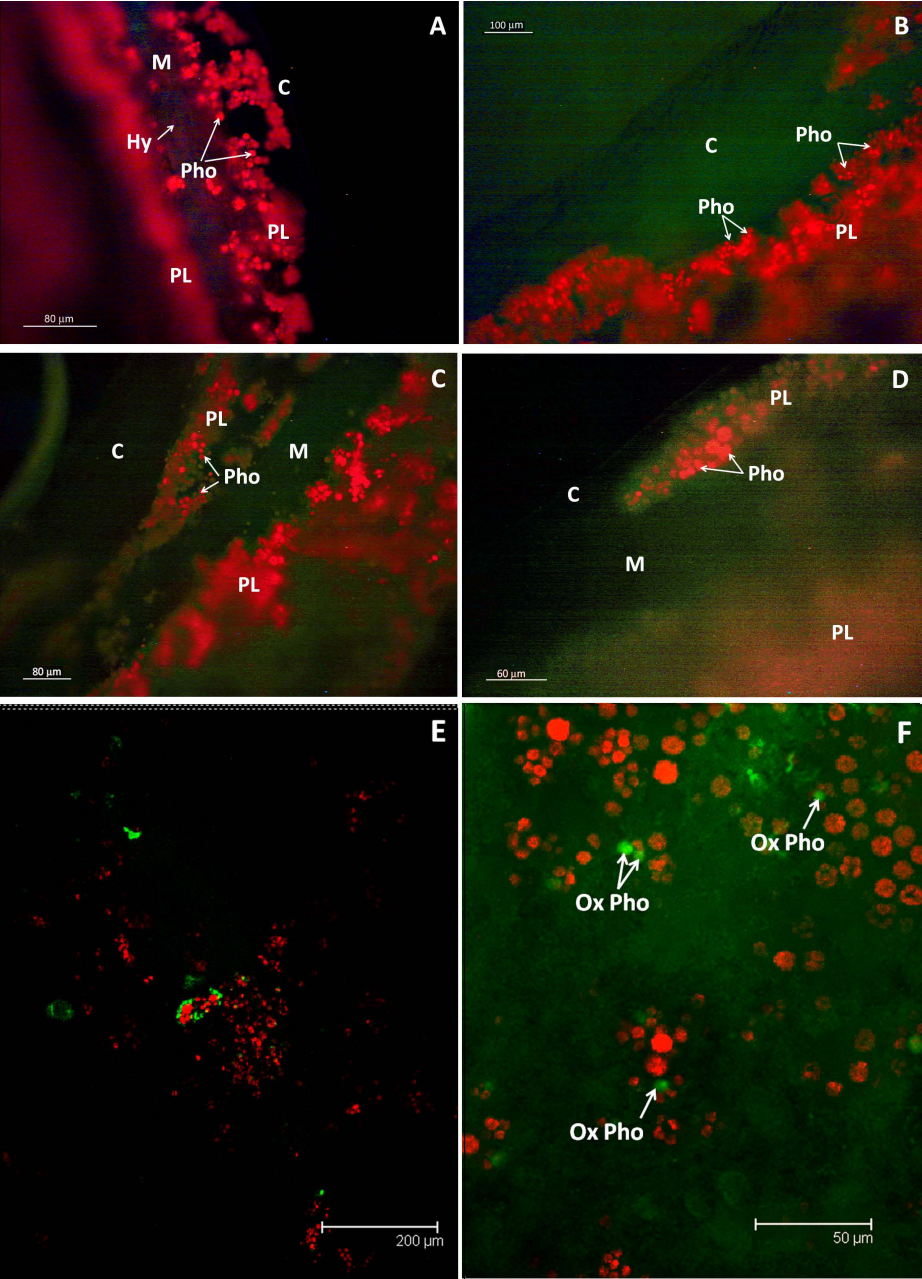
**ROS in rehydrated *R. farinacea *thalli**. Thalli of *R. farinacea *rehydrated with deionized water and 10 μM DCFH_2_-DA and observed 3-4 h post-rehydration. **A, B, C, D **ROS content, as revealed by the green fluorescence emission of DCF under a fluorescence microscope (magnification: 400× for A, B and 1000× for C, D); **E **overlay of confocal microscopy images reveals ROS distribution around some of the photobionts (green fluorescence); **F **overlay of confocal microscopy images of ROS content of *R. farinacea *thalli that had been rehydrated with c-PTIO 200 μM, arrows point to photobionts photobleached by the confocal laser during the observation (oxPho). Red fluorescence is due to the photobiont's chlorophyll in all cases. Each micrograph is representative of several images corresponding to independent samples. C cortex, M medulla, PL photobiont layer, Pho photobiont, oxPho photobleached photobiont, Hy fungal hyphae

The presence of 2',7'- dichlorofluorescin (DCF), the fluorescent oxidation product of DCFH_2_, indicated the intracellular production of free radicals during lichen rehydration (Figure 2B-D). DCF was especially concentrated in the lichen cortex. No significant green autofluorescence was detected in the absence of the probe (Figure 2A). Confocal microscopy showed discrete points of green fluorescence around several large photobionts (Figure 2E), probably due to mycobiont hyphae tips.

**Figure 3:**
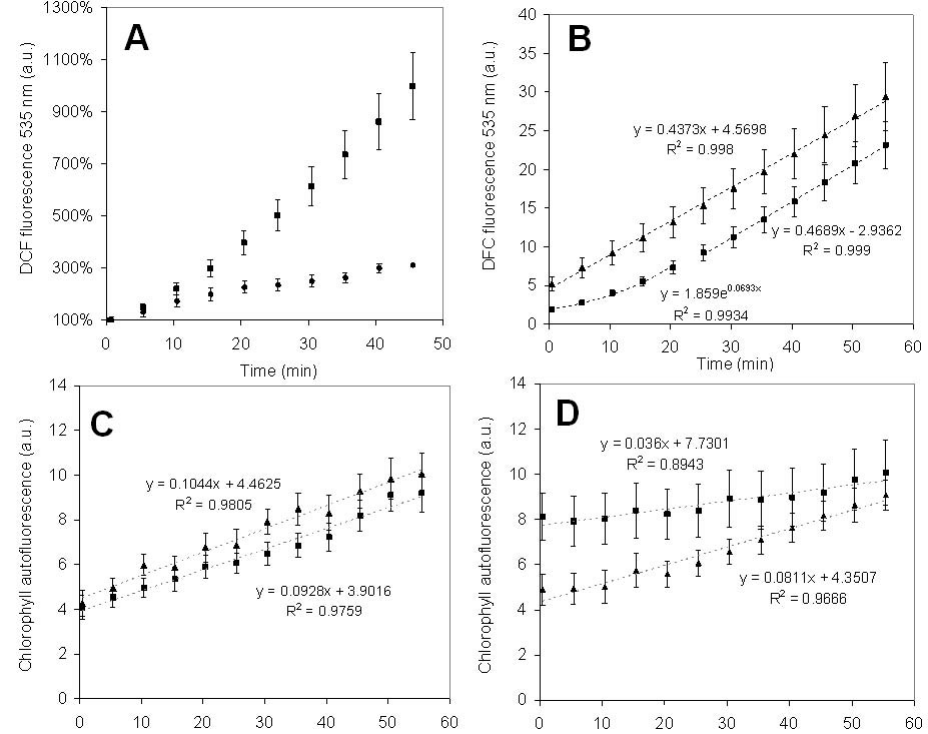
**Fluorometric kinetics of free radical production and chlorophyll autofluorescence in *R. farinacea *thalli**. **A**, Kinetics of intracellular free radical production evidenced by DCF fluorescence in recently rehydrated thalli (solid squares) compared with thalli hydrated for 24 h (solid circles); **B**, Kinetics of intracellular free radical production evidenced by DCF fluorescence in thalli rehydrated with deionised water (solid squares) or c-PTIO 200 μM (solid triangles); **C, **chlorophyll autofluorescence in lichens rehydrated with deionised water (solid squares) or c-PTIO 200 μM (solid triangles); **D, **chlorophyll autofluorescence in thalli hydrated 24 h before, and treated for 5 min with deionised water (solid squares) or c-PTIO 200 μM (solid triangles). Fluorescence units are arbitrary and comparisons of relative magnitudes can only be made within the same graph. Bars represent means and error bars the standard error of 12 replicates.

A fluorometric kinetics of intracellular free radical production in *Ramalina farinacea *thalli was performed in order to confirm microscopical data. Figure 3A demonstrates that the rate of intracellular free radical production in recently rehydrated thalli was much higher than the rate of intracellular free radical production in thalli kept in the hydrated state during the previous 24 h. Furthermore, intracellular release of free radicals during rehydration under physiological conditions was biphasic with an initial exponential phase of 20 min followed by a linear phase (Figure 3B). Chlorophyll autofluorescence was simultaneously recorded since this parameter is a surrogate of the levels and integrity of this molecule and therefore of the photosynthetic status of the cell. A slight increase in chlorophyll autofluorescence both in thalli hydrated for 24 h (Figure 3C, solid squares) and in thalli recently rehydrated (Figure 3D, solid squares) could be measured.

**Figure 4:**
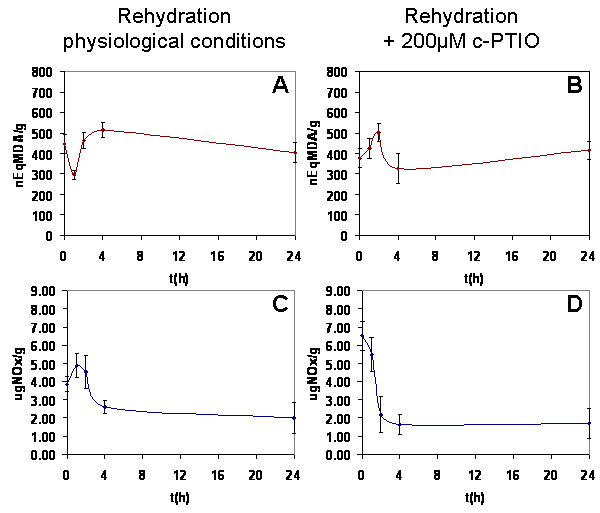
**MDA content and NO end-products of rehydrated *Ramalina farinacea *thalli**. MDA content: **A **rehydration with deionized water, **B **rehydration with c-PTIO (200 μM) in deionized water. NO end-products: **C **rehydration with deionized water, **D **rehydration with c-PTIO (200 μM) in deionized water. Student *t *test: * p < 0.05. The error bars stand for the standard error of at least 9 replicates

To determine whether the observed increase of ROS caused oxidative stress during rehydration, lipid peroxidation in *R. farinacea *was quantified in the first 24 h of rehydration under physiological conditions. After 1 h of rehydration, MDA levels dropped significantly to a minimum (Figure 4A). After 2 h, the levels began to increase such that they were slightly elevated at 4 h, at which time the maximum value was reached. This latter amount was unchanged at 24 h post-rehydration.

### NO release during lichen rehydration

The release of NO in a lichen species was recently demonstrated for the first time. In order to confirm these results in another lichen species, *R. farinacea*, two approaches were used: fluorescence visualization of the released NO and quantification of the NO end-products. Accordingly, thalli were rehydrated in deionized water containing 200 μM DAN for the visualization of NO release and in deionized water alone for the quantification of NO end-products.

**Figure 5:**
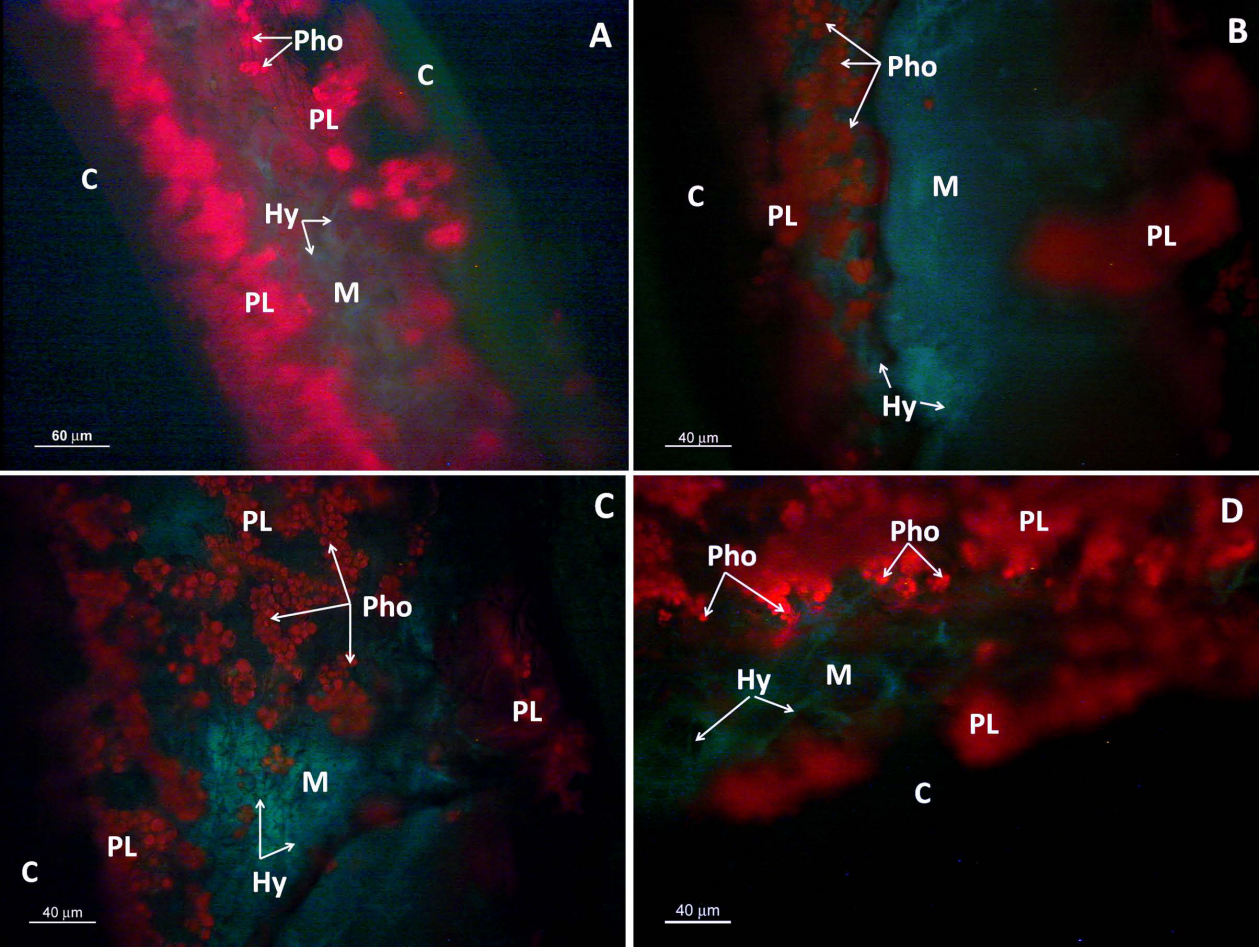
**NO content of rehydrated *R. farinacea *thalli**. Fluorescence microscopy of thalli of *R. farinacea *rehydrated with deionized water and 200 μM DAN. Blue fluorescence evidence NO presence, red fluorescence is due to the photobiont's chlorophyll in all cases. Micrographs are representative of several images corresponding to independent samples. C cortex, M medulla, PL photobiont layer, Pho photobiont, Hy fungal hyphae

Microscopic analysis of blue fluorescence evidenced the production of NO, which was intimately associated with the fungal hyphae. Staining was especially intense in the medulla (Figure 5).

Air oxidation of NO in an aqueous environment results in the near exclusive generation of NO_2_^-^, which is further oxidized to NO_3 _^= ^[23]. NO end-products (NO_x_) were quantified by the classical method of Griess. NO_x _levels increased over 2 h to reach a maximum (Figure 4C). By 4 h, NO_x _levels had decreased to slightly below the initial levels, reaching a minimum, after which the levels remained constant for up to 24 h.

### Effect of NO scavenging during lichen rehydration on ROS production, chlorophyll autofluorescence and lipid peroxidation

To study the role of NO during rehydration, *R. farinacea *thalli were rehydrated with 200 μM of the membrane-permeable compound c-PTIO, which specifically reacts with NO to inhibit its biological actions.

NO scavenging with c-PTIO completely suppressed DAN fluorescence emission (image not shown). It also produced a remarkable increase in ROS production in both the cortex and the medulla (Figure 2F). The confocal laser beam produced an oxidative burst in the photobionts, leading to chlorophyll photo-oxidation and DCF fluorescence onset within seconds (Figure 2F).

The kinetics study (Figure 3B, solid triangles) confirmed that NO inhibition during rehydration multiplies the levels of intracellular free radicals at 0 min (52.1 ± 2.85 versus 18.4 ± 1.67 a.u.). Moreover, inhibition of NO eliminates the initial exponential phase of free radical production seen during physiological rehydration of thalli (Figure 3B, solid squares). Chlorophyll autofluorescence was simultaneously measured and no evident differences between physiological and NO-inhibited rehydration could be observed (Figure 3C, solid triangles). However, NO inhibition in 24h-hydrated thalli resulted in an important decrease in chlorophyll autofluorescence that tends to recover normal values after 1 h (Figure 3D, solid triangles).

Lipid peroxidation during NO-specific inhibition with c-PTIO was measured quantitatively; the results are presented in Figure 4B. MDA levels reach a maximum at 2 h and a minimum at 4 h. The MDA levels measured following rehydration with cPTIO were the opposite of those obtained under physiological conditions. Figure 4D shows that, overall, NO end-products decreased in amount when c-PTIO was used.

### Microscopy studies of isolated algae

Confocal studies clearly showed that NO deprivation caused photo-oxidative damage in the photobiont (Figure 2F). NO is known to reduce photo-oxidative stress in some species of green algae. A specific role for NO in the prevention of photo-oxidation in *Trebouxia *algae was confirmed in the following studies.

**Figure 6:**
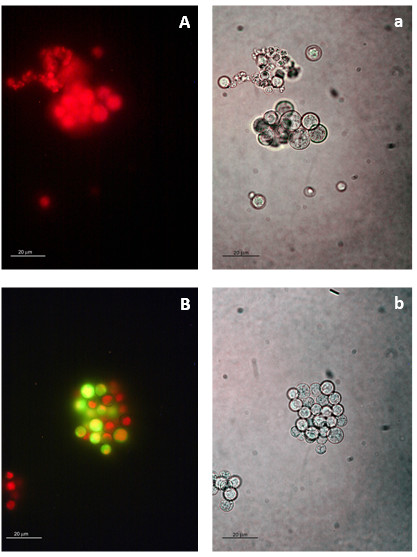
**ROS content of isolated *Trebouxia *sp**. Capital letters identify the fluorescence image; the lower-case letter indicates the corresponding bright-field images: A-a control; B-b algae treated with 200 μM c-PTIO. Each micrograph is representative of several images corresponding to independent samples. Magnification 1000×. Bar 20 μm

A suspension of axenically cultured *Trebouxia *sp., the photobiont isolated from *R. farinacea*, was treated with 200 μM c-PTIO in the presence of both DCFH_2_-DA and DAN. The images of control cells are presented in Figure 6A. NO inhibition by c-PTIO resulted in chlorophyll bleaching in some algae (Figure 6B). DAN fluorescence could not be detected by this method but the oxidative burst caused by c-PTIO provided indirect evidence of endogenous NO production in the algae. Direct measurements of NO end-products in the supernatant of photobiont suspensions at different time periods of culture (0-24 h) showed that these algae were able to produce NO in the low-nanogram range. NO levels reached a peak of 567 ng per million cells 2 h after preparation of the suspension (Table 1).

**Table 1:** NO end-products of the Trebouxia sp. photobiont isolated from Ramalina farinacea at different time points after the establishment of the algal suspension

Time (h)	ng NO_x_/10^6 ^cells ± standard error (n = 9)
0	3.87 ± 0.378
1	3.49 ± 0.418
2	567 ± 282
4	3.17 ± 0.461
24	3.06 ± 0.414

### Photosynthetic studies on isolated algae

**Figure 7:**
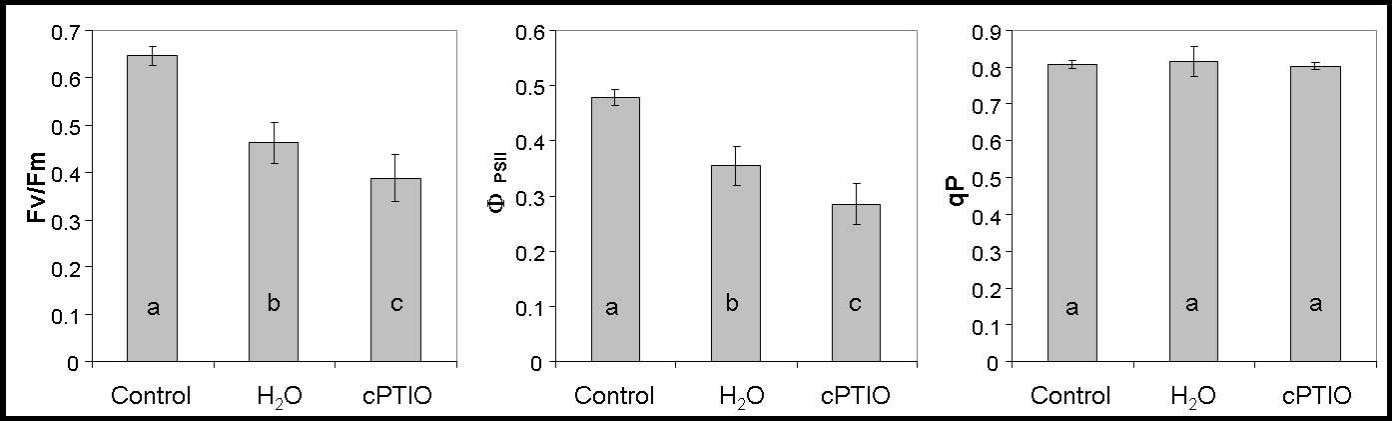
**Effect of NO inhibition in *Asterochloris erici *photosynthetic parameters**. Photosynthetic parameters of axenic cultures of *Asterochloris erici *desiccated for 24 h and then rehydrated with either deionized water or 200 μM c-PTIO. The algae were incubated under normal culture conditions for 24 h before chlorophyll *a *fluorescence was measured. Control algae were not desiccated but instead maintained under normal culture conditions. F_v_/F_m_, maximum photochemical efficiency of photosystem II (PSII); Ф_PSII_, photochemical efficiency in light; qP, photochemical component of fluorescence relaxation. Different letters show significant differences between treatments. LSD test (*p *< 0.05), *n *= 3

To confirm that the visualized alterations in chlorophyll fluorescence were linked to alterations in the photosynthetic activity of the algae during NO deprivation, axenic cultures of *Asterochloris erici*, a well-characterized photobiont, were studied. The cells were cultured on cellulose-acetate discs, desiccated for 24 h, and rehydrated with 200 μM c-PTIO. Measurements were made in cells that had been maintained in culture conditions for 24 h. The significant decrease of F_v_/F_m _and Ф_PSII _indicated that NO scavenging induces photo-inhibition of PSII (Figure 7). The degree of quinone A (Q_A_) oxidation was determined as qP, which depends on the activation state of photosystem I (PSI) and the Calvin cycle [36]. After the dehydration/rehydration cycle, no differences were observed in qP, indicating that photoinhibition was produced before Q_A_.

The same treatments and measurements were carried out in whole thalli of *R. farinacea *but no alterations in photosynthesis at 24 h were observed (data not shown).

## Discussion

This study investigated the role of NO during rehydration in *Ramalina farinacea*. The results showed that lichen NO plays an important role in the regulation of lipid peroxidation and photobiont photo-oxidative stress during rehydration.

NO is a well-studied critical signaling molecule involved in abiotic stress responses [14] and plant defence [13]. Our results demonstrated that, in addition to its utility for quantification methods, DAN is an excellent fluorescence microscopy probe for the histophysiological characterization of NO production in lichen.

The ability of ROS production to induce oxidative stress depends on the balance between cellular pro-oxidants and antioxidants, with an imbalance between the two resulting in oxidative damage. Thus, studies of ROS release using probes such as DCFH_2 _only determine the levels of pro-oxidant species but do not indicate the degree of oxidative stress. Instead, lipid peroxidation, measured as MDA, has long been used to characterize oxidative damage in cells and was the approach used in this study.

Our data showed that rehydration is accompanied by ROS and NO generation and thus confirmed the results of Weissman et al. [20]. The kinetics of ROS release is biphasic with an initial exponential phase (20-30 min) followed by a linear phase up to 1 h. The quantification of NO end-products showed that released NO reaches a maximum 1-2 h post-rehydration. Despite the presence of ROS, lipid peroxidation significantly decreased during the first hours following rehydration, reaching a minimum after 2 h, which coincided with the maximum levels of NO end-products.

Our microscopy studies revealed that the production of ROS and NO is closely related to lichen morphology: ROS was mainly associated with the hyphae of the cortex whereas NO was clearly localized to the medullar hyphae of the mycobiont. Confocal microscopy confirmed that the medulla is free of intracellular ROS, which were seen only in a few punctate zones around several large photobionts (Figure 1C). Since ROS are now recognized as key signaling molecules in yeast and in plants [14,15,37], these areas could constitute points of communication between the fungus and algae and are perhaps related to the mutual up-regulation of protective systems, as suggested by Kranner et al. [5]. Further investigations are needed to clarify this point.

NO scavenging during lichen rehydration resulted in increased ROS production and lipid peroxidation. Moreover, the initial exponential phase of free radical production is eliminated. This finding demonstrates that NO is involved in antioxidant defense and the regulation of lipid peroxidation especially during the first minutes after rehydration. In plants and in animals, NO is known to modulate the toxic potential of ROS and to limit lipid peroxidation, acting as a chain-breaking antioxidant to scavenge peroxyl radicals [12,16,38].

The incidence of the confocal laser on the algae of NO-deprived rehydrating thalli caused a rapid photo-oxidative burst and isolated photobionts showed evidence of oxidative destruction of chlorophyll even in the absence of the photo-stress caused by a confocal laser. Furthermore, NO-endproducts quantification supports the ability of *Trebouxia *photobionts to produce NO, eventually in important amounts (Table 1). Chlorophyll autofluorescence informs about the levels and integrity of this molecule. No appreciable changes in chlorophyll autofluorescence were seen during rehydration but the inhibition of NO in thalli hydrated for 24 h induced a reversible decrease in this parameter during 1 h. NO has been shown to ameliorate ROS toxicity in the chlorophycean alga *Scenedesmus obliquus*, probably by preventing the photo-inhibition that leads to photo-oxidation and pigment bleaching [39]. Our studies on the physiology of photosynthesis show that the inhibition of NO action altered the photosynthetic activity of the photobionts. These results suggest that NO is involved in PSII stabilization and could be related with the limited role of classical antioxidant systems during desiccation-rehydration cycles in *Asterochloris *(formerly *Trebouxia*) photobionts recently reported [7]. Several authors have demonstrated that, in higher plants, NO reversibly binds to PSII [40-44] and modulates electron transfer and quenching processes [45]. The fact that the same dose of c-PTIO than that used for photobionts did not alter photosynthetic activity in the photobionts of intact lichens suggests that the mycobiont is involved in stabilizing the photobiont's chlorophyll. Assays with higher doses of c-PTIO and specific inhibitors of fungal NO synthases are needed to confirm this possibility.

## Conclusions

**Figure 8:**
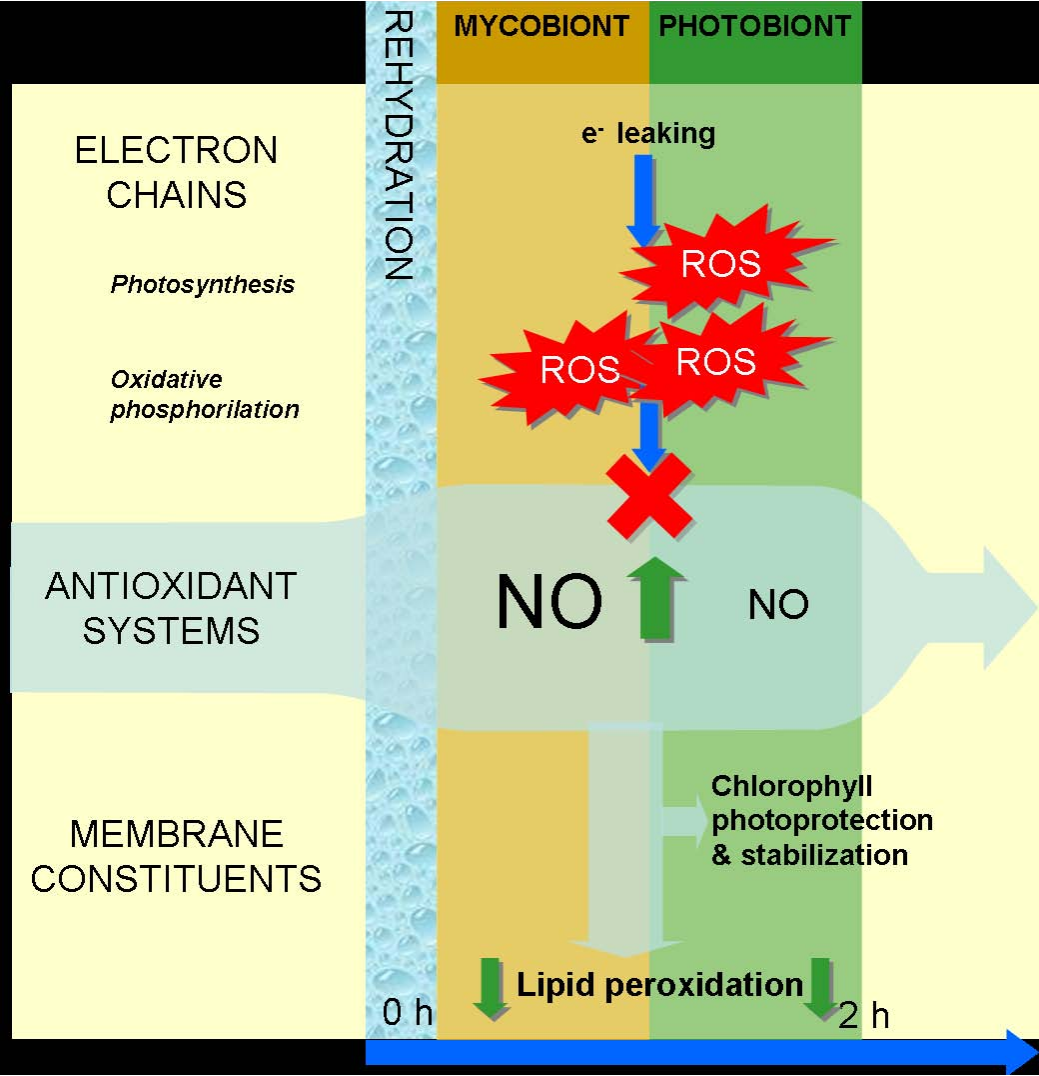
**Schematic representation of the findings of the present work on the functional relation of nitric oxide (NO) with oxidative stress during rehydration of *Ramalina farinacea *in the context of current knowledge**. Rehydration induces the functional reconstitution of electron chains, the most relevant being chloroplast photosynthesis and mitochondrial oxidative phosphorilation. During the process of reconstitution, membrane molecular architecture is not optimal and an elevated electron leaking from electron chains occurs. Electron leaking causes a burst of intracellular ROS. Nitric oxide is released mainly from mycobiont medular hyphae (NO production by photobionts has not been confirmed in the lichen but is likely). A decrease in lipid peroxidation of lichen thalli coincides with the peak of NO-endproductos. NO participates in chlorophyll photoprotection and stabilization during rehydration

These data provide the first evidence of an important role for NO in oxidative stress regulation during the early stages of rehydration in the lichen *Ramalina farinacea*, including chlorophyll photostability of the trebouxioid photobionts (summarized in Figure 8). Our results also raise important questions about the evolutionary role of NO in the establishment of lichen symbiosis, due to its dual role as antioxidant and mediator in cell communication.

Future research should be directed at functional (NO donors, NO synthase inhibitors, exposure to SO_2_, Cu^+/2+^, etc.), ultrastructural (sites of NO synthesis, immunohistochemistry), and cell communication (co-culture of isolated symbionts, NO donors, c-PTIO) studies of NO, with the aim of clarifying the role of this multifaceted molecule.

## Abbreviations

a.u.: arbitrary units; BHT: 2,6-di-tert-buthyl-4-methylphenol; c-PTIO, carboxy-PTIO: 2-(4-carboxyphenyl)-4,4,5,5-tetramethylimidazoline-1-oxyl-3-oxide, potassium salt; DAN: 2,3-diaminonaphthalene; DCF: 2',7'- dichlorofluorescin; DCFH_2_: 2',7'- dichlorodihydrofluorescein; DCFH_2_-DA: 2',7'- dichlorodihydrofluorescein diacetate; MDA: malondialdehyde; NAT: 1-H-naphthotriazole; ROS:reactive oxygen species; TEP: 1,1,2,2-tetraethoxypropane

## Authors' contributions

EB and MC conceived Objectives and designed the study and general design of the work. FG and EB collected and identified *R. farinacea *thalli. Microscopy and image handling were performed by FG-B and J R-A. FG designed and carried out photobionts isolation and physiology of photosynthesis experiments. Studies on lipid peroxidation and NO-endproducts quantification were made by AEP. MC and FG wrote the paper and EB made final considerations. All authors read and approved the final manuscript.
